# A systematic review of malignancy-associated hemophagocytic lymphohistiocytosis that needs more attentions

**DOI:** 10.18632/oncotarget.19230

**Published:** 2017-07-14

**Authors:** Hongluan Wang, Lixia Xiong, Weiping Tang, Ying Zhou, Fei Li

**Affiliations:** ^1^ Department of Hematology, The First Affiliated Hospital of Nanchang University, Nanchang, Jiangxi 330006, China; ^2^ Medical College, Nanchang University, Nanchang, Jiangxi 330006, China; ^3^ Department of Respiratory, Jiangxi Provincial People's Hospital, Nanchang, Jiangxi 330006, China

**Keywords:** hemophagocytic lymphohistiocytosis, hemophagocytic syndrome, malignancy, lymphoma

## Abstract

As an infrequent but potentially life-threatening hyperinflammatory syndrome, hemophagocytic lymphohistiocytosis (HLH) is clinically characterized with prolonged fever, hepatosplenomegaly, cytopenia, hypertriglyceridemia, hyperferritinemia and hemophagocytosis in bone marrow, liver, spleen or lymph nodes. Malignancy-associated HLH (M-HLH), one type of acquired HLH, usually presents variable overlaps of symptoms with other types of HLH, thus resulting in higher incidence of misdiagnosis and mortality. In recent years, with the increasing awareness to this disease, the diagnosis and management of HLH have gained more and more attention, and improvements have been made accordingly. As a result, the survival of patients is greatly prolonged. However, there is still no consensus on the diagnostic criteria and treatment strategies due to lack of large samples or prospective clinical trials. In order to improve recognition and diagnosis, and provide guidance regarding the treatment of M-HLH, the Study Group in HLH Subtypes of the Histiocyte Society has developed consensus recommendations for the diagnosis and management of M-HLH in 2015. In the present article, we summarized and discussed some updated understandings in M-HLH.

## INTRODUCTION

Hemophagocytic lymphohistiocytosis (HLH), also called hemophagocytic syndrome (HPS), is an infrequent but potentially life-threatening hyperinflammatory syndrome, including impaired function of cytotoxic T lymphocytes and natural killer (NK) cells, as well as macrophages [1]. The clinical characteristics of HLH are complicated, generally including prolonged fever, hepatosplenomegaly, cytopenia, hypertriglyceridemia, hyperferritinemia and hemophagocytosis in bone marrow, liver, spleen or lymph nodes. Primary HLH consists of several genetic conditions, including familial-HLH (F-HLH) 2-5, Griscelli syndrome type II, Chediak-Higashi syndrome and other types. HLH is mainly triggered by infections, and the onset of primary HLH usually occurs during childhood although reports regarding its adult presentation are increasing [2]. Secondary or acquired HLH is caused by a wide range of causes, including infections, malignancies, autoimmune diseases, metabolic diseases and acquired immune deficiencies (such as AIDS, iatrogenic immune suppression and organ or stem cell transplantation) [3]. In the past 10 years, more than 1,500 publications have appeared with the increasing awareness to this disease [4]. Of these, HLH in the context of malignancy is considered as a big challenge to clinicians due to variable overlaps of symptoms with other types of HLH, sepsis and multiorgan failure, thus resulting in higher incidence of misdiagnosis and mortality [3]. Prompt diagnosis and treatment are crucial to initiate appropriate treatment, which can avoid a fatal outcome caused by multiorgan dysfunction. However, only very few reports currently focus on the malignancy-associated HLH (M-HLH) due to low incidence and insufficient knowledge. Therefore, we reviewed some key points, including epidemiological data, predisposing factors, diagnosis, treatment strategies and prognosis of M-HLH in this article, and our study provided the valuable guidance for the treatment of M-HLH patients.

### Epidemiological profile

**Figure 1:**
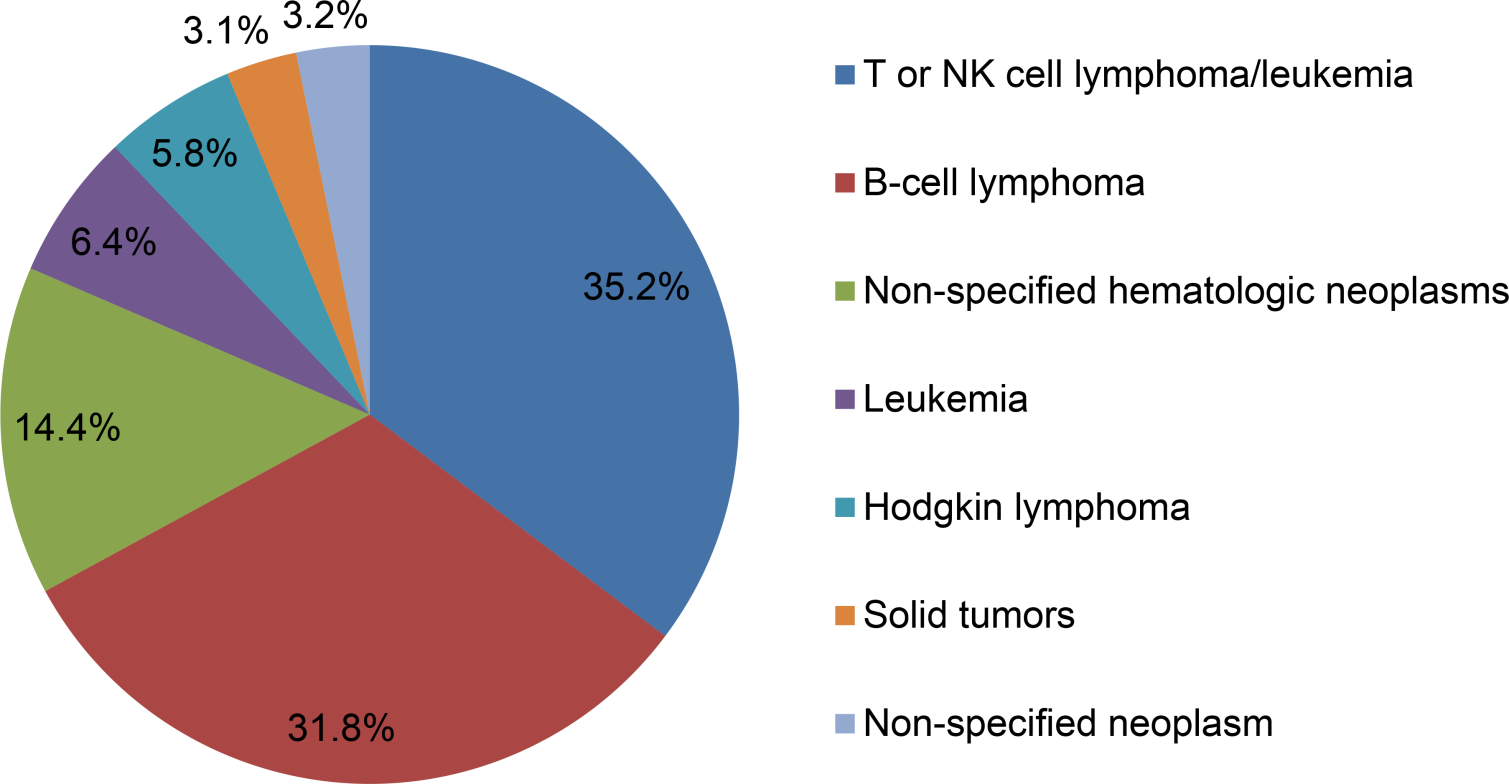
The tumor types of malignancy-associated HLH

HLH is a rare disease, but it presents an increasing incidence in recent years. Currently, the accurate epidemiological profile of HLH is not well defined. Some surveys in Italy, Sweden and USA have reported that the estimated annual incidence of HLH is approximately 800,000 people and 1–10 per 1 million children [5–7]. Similarly, the epidemiological data in M-HLH patients also remain limited. In recent years, patients with hematologic neoplasms, especially lymphoma, are prone to HLH, which has gained more and more attention by clinicians. HLH affects 1% of adults with hematologic tumor, but the incidence is increased to 20% in some patients with B- and T-cell lymphomas [8]. A large series of studies from 2,197 adult HLH patients have demonstrated that M-HLH accounts for approximately 50% of adult HLH. The most common tumor types for triggering HLH are hematological neoplasms (93.7%) with T- or NK cell lymphoma or leukemia (35.2%), followed by B-cell lymphoma (31.8%), other non-specified hematologic neoplasms (14.4%), leukemia (6.4%) and Hodgkin lymphoma (5.8%). Solid tumors and non-specified neoplasms account for 3.1% and 3.2%, respectively [9] [Figure 1]. In T-cell malignancies, peripheral T-cell lymphoma, primary cutaneous γδ-T-cell lymphoma, anaplastic large cell lymphoma and lymphoblastic lymphoma are more likely to trigger HLH [10, 11]. In addition, diffuse large B-cell lymphoma (DLBCL) is the most frequent B-cell lymphoma that can trigger HLH. B-precursor acute lymphoblastic leukemia is sporadically reported, which is the most common childhood tumor [12]. No specific predominant subtype is reported in Hodgkin lymphoma [13]. Other malignant or non-malignant hematologic diseases associated with HLH include Epstein-Barr virus (EBV)-associated T/NK-cell lymphoproliferative disorders, leukemia, myelodysplastic syndrome, Langerhans cell histiocytosis, multicentric Castleman disease and cytophagic histiocytic panniculitis [14–17]. Solid tumors, such as Wilms tumor [18] and germ cell tumors [19], are sporadically reported with the incidence of only 3% in adult HLH patients [9]. Geographical variability is another epidemiological characteristic of HLH patients. DLBCL-triggered HLH is predominant in Western countries and Japan [20, 21], while T-cell neoplasm is the major trigger of HLH in China and Korea [22, 23], suggesting that specific genetic backgrounds affect the distribution of cause for HLH patients [10]. Table 1 lists some types of M-HLH.

**Table 1:** Previously reported malignancy-associated HLH

Malignancy-associated HLH	Reported cases
**Malignancy-triggered HLH**	
T-cell or NK-cell lymphoma	Peripheral T-cell lymphoma (Unspecified) [24] NK/T cell lymphoma, aggressive NK cell leukemia, gastric T-cell lymphoma [24] Anaplastic large cell lymphoma, ALK negative [25] Anaplastic large cell lymphoma, ALK positive [26] Angioimmunoblastic T-cell lymphoma [27] Panniculitis-like T-cell lymphoma [28] Gamma-delta (γδ) T-cell lymphoma [29] Lymphoblastic lymphoma/leukemia, Hepatosplenic T-cell lymphoma [30]
B-cell lymphoma	Diffuse large B-cell lymphoma [31] T-cell/histiocyte-rich large B-cell lymphoma [32] Follicular lymphoma, marginal zone lymphoma, intravascular large B-cell lymphoma, Burkitt's lymphoma [30]
Hodgkin lymphoma	Lymphocyte-depleted Hodgkin lymphoma [13]
Not specified lymphoma	Primary bilateral adrenal lymphoma [33]
Leukemia	Acute lymphoblastic leukemia (B-cell precursor acute lymphoblastic leukemia, T-cell acute lymphoblastic leukemia) [34, 35] Acute myelocytic leukemia [36] Acute monoblastic leukemia [37] Acute erythroid leukemia [38] Acute megakaryocytic leukemia [39] Chronic lymphocytic leukemia [40]
Other hematological neoplasms	Castleman's disease [41] Myelodysplastic syndromes [42] Multiple myeloma [9]
Solid tumor	Wilms tumor [18] Germ cell tumor [19] Lung cancer, prostate cancer, hepatocellular carcinoma [43] Colonic malignancy [28] Squamous cell carcinoma of the neck [44]
Not specified neoplasm	Mediastinal endodermal sinus tumor [45]
**HLH during chemotherapy**	
	Acute myeloid leukemia [46] Acute promyelocytic leukemia [47] Chronic lymphocytic leukemia [48] T-cell acute lymphoblastic leukemia [49] Classical Hodgkin lymphoma [49] Neuroblastoma [20]

HLH can be observed at any age, but the probability to develop HLH in patients with lymphoma may increase with the age. A large survey with 799 HLH patients in Japan indicates that lymphoma is detected in 68% of patients over 60 years old, 38% of patients within 30–59 years old, 10% of patients within 15–29 years old, and 0% of patients under 14 years old [21]. However, another report has shown that M-HLH is observed in pediatric and adolescent patients with the prevalence of approximately 8% [49]. In addition, Hodgkin lymphoma has been presented in several types of hereditary HLH [50]. Booth et al. have reported that B-cell non-Hodgkin lymphoma (NHL) occurs in 24% of patients with X-linked lymphoproliferative syndrome type I, a disorder associated with aggressive HLH, due to *SAP/SH2D1A* deficiency [51]. Moreover, malignancies have also been reported in patients with F-HLH 2, 4 and 5, who are detected to have hypomorphic alleles in *PRF1*, *STX11* and *STXBP2* [52].

### Predisposing factors

Malignant cells or/and infections, such as viruses, invasive fungi and bacteria, are the major triggers or co-triggers contributing to the secretion of excessive cytokines and the development of HLH, such as EBV-associated lymphoma. HLH can occur during the phase of onset or relapsed malignancies, also during the phase of chemotherapy, including induction, consolidation and even maintenance therapy due to therapy-induced immunosuppression, which usually occurs in the treatment of lymphoma or leukemia.

Malignant or infected cells can initiate the immune response. Dysfunctional cytotoxic CD8+ T lymphocytes (CTLs) and NK cells are unable to initiate a proper response against the target cells. This results in an uncontrolled proliferation of the CTLs, a large production of interferon-γ (INF-γ) and proliferation of histiocytes (macrophages) that subsequently invade organs, such as liver, spleen and lymph nodes, and produce a further storm of cytokines, including INF-γ, TNF-α, and interleukins (IL)-1, 6 and 18 [5]. The proliferating histiocytes engulf red cells, white cells, platelets and are called hemophagocytes.

More attention should be paid to the correlation among EBV infection, lymphoma and HLH. As one of the herpes groups, EBV infects more than 95% of the adult population worldwide and is the most frequent infective trigger of HLH. Many types of lymphoma, such as T/NK cell lymphoma, have been identified to be related to EBV infection. Its transmission occurs predominantly through exposure to infected saliva. EBV has a well-described tropism for B cells, and the invasion of CTL and NK cell populations plays an important role in the pathogenesis of HLH. Abnormal cytotoxic activity prevents efficient removal of infected cells, leading to continuous antigenic stimulation and dysfunctions of CTLs and NK cells and finally resulting in life-threatening hyperinflammatory syndrome and HLH.

### Diagnosis and differential diagnosis

The clinical presentations, signs and laboratorial abnormalities of HLH are diverse, mainly including continuous high fever (> 38.5°C), hepatosplenomegaly, cytopenia, skin rashes, panniculitis-like cutaneous nodules, multiple involvement of internal organs, increased lactate dehydrogenase (LDH), hypertriglyceridaemia, hyperferritinaemia, disseminated intravascular coagulopathy and high concentrations of soluble CD25 or CD163. The diagnostic criteria for HLH are in accordance with the guideline proposed by the Histiocyte Society in 1991 and updated in 2004. Table 2 lists the diagnostic criteria of HLH-2004.

**Table 2:** HLH-2004 diagnostic criteria [53]

**• Genetic defect consistent with HLH** **• Fulfillment of five of the eight following clinical criteria:**	
1. Fever	Temperature > 38.5°C for > 7 days
2. Splenomegaly	Spleen tip palpated > 3 cm below left costal margin
3. Cytopenia	≥ 2 lineages
Hemoglobin	< 90 g/L (neonates < 100 g/L )
Platelets	< 100 × 10^9^/L
Neutrophiles	< 1 × 10^9^/L
4. Hyperferritinemia	> 500 μg/L
5. Hypofibrinogenemia or hypertriglyceridemia	< 1.5 g /L, or > 3 mmol/L
6. Elevated soluble CD25	> 2,400 U/mL
7. Hemophagocytosis	Bone marrow, spleen, liver, lymph node or other tissues
8. Reduced or absent NK cytotoxicity	
**Supportive evidence**	
Elevated transaminases and bilirubin	
Elevated lactate dehydrogenase	
Elevated d-dimers	
Elevated cerebrospinal fluid cells and/or protein	

NK: natural killer cells.

The diagnosis of M-HLH is particularly challenging because the symptoms are nonspecific and many symptoms overlap among some severe illnesses, including sepsis, systemic inflammatory response syndrome (SIRS), multiorgan failure and hematologic malignancies. So far, there are no universally accepted diagnostic criteria for M-HLH. It still remains controversial whether the widely used HLH-2004 criteria are suitable for M-HLH patients, because the first criteria defined in the 1990s were based on the pediatric patients from the international treatment HLH-94 study and expert opinions modified according to the subsequent HLH-2004 study [53]. Other diagnostic indicators have not gained the wide acceptance. For example, Takahashi et al. [54] have proposed to add LDH and d-dimers into the diagnostic criteria based on 142 cases with adult lymphoma-associated HPS (LAHS). Maruoka et al. [55] have identified IP-10/CXCL10 and MIG/CXCL9 as novel markers for the diagnosis of LAHS using cytometric bead array (CBA) with sensitivity and specificity of 100% and 95%, respectively. Furthermore, IP-10 and MIG have been used to distinguish LAHS from sepsis in patients with hematologic malignancies. In addition, EBV is a frequent co-trigger in 24% of M-HLH patients and in 88% of patients with HLH during chemotherapy (C-HLH) [49]. In spite of the limitations, the HLH-2004 criteria are still universally accepted as a substitute definition [56]. Patients with five of eight criteria and potential malignancy can be diagnosed as M-HLH.

Lymphoma should be considered as a possible underlying disease in any HLH patients. It is also suitable for patients with obvious EBV, CMV or HIV infection. It is well known that EBV infection as a co-trigger is observed in 90% of Hodgkin lymphoma and approximately 33% of peripheral T-cell lymphoma [20, 57]. Lymphoma can be detected in more than 50% of adult HLH patients with HIV infection [58]. In addition, in patients with immunity-associated HLH, such as systemic lupus erythematosus, systemic juvenile idiopathic arthritis or adult-onset Still's disease (AOSD), a careful search of a malignancy should be recommended to avoid any missing diagnosis of hidden neoplasms. Under some hereditary conditions predisposing to HLH, malignancy, especially lymphoma, should also be excluded.

Previous studies have reported that several indicators reflect the severity of diseases, which can be used to further screen the possible M-HLH patients. Soluble IL-2 receptor (sCD25) is a valuable marker with constantly increased level during active HLH. In addition, a high ratio of CD25 to ferritin is more often detected in LAHS and may be a useful marker to distinguish other types of HLH [59]. Moreover, we have retrospectively analyzed the clinical characteristics, laboratorial findings and survival time in 16 LAHS patients from 69 adult HLH patients, and found that LAHS patients have obviously lower levels of fibrinogen (Fbg, < 1.5 g/L) and platelet (PLT, < 40 × 10^9^/L), higher levels of LDH (≥ 1,000 U/L) and the worst survival compared with other types of HLH [24]. These characteristics may offer more likelihood for us to screen potential lymphoma. As for these suspicious patients, X-ray of the chest and ultrasound or computed tomography of the abdomen should be recommended to screen enlarged lymph nodes. Suspicious lymph nodes or cutaneous lesions should be biopsied. If it is possible, magnetic resonance imaging (MRI) or positron emission tomography (PET) may be helpful for the diagnosis in some cases.

Other severe illnesses, including sepsis, SIRS and multiorgan failure, usually share similar clinical symptoms and laboratorial abnormalities as HLH, making the differentiation more complicated. Moreover, two diagnostic indicators (high concentration of soluble CD25 and decreased NK-cell activity) are also reported in sepsis, SIRS and multiple organ dysfunction syndrome (MODS), suggesting that these diseases may share some common mechanisms [9]. Hyperferritinemia may be a major indicator in the differentiation of HLH from other systemic diseases. Allen et al. [60] have assessed 330 patients with high ferritin levels (greater than 500 μg/L), and 10 patients are diagnosed as HLH. Ferritin, as an indicator, is 90% sensitive and 96% specific for HLH when its level is over 10,000 μg/L. However, the elevated ferritin must be differentiated from transfusion-related iron or malignancy-related overload.

### Treatment

The treatment of M-HLH aims to control the overactive immune system and treat malignancy. However, there are no universal conclusions on whether an HLH-directed, malignancy-directed or combined approach should initially be adopted due to lack of prospective, randomized or controlled clinical trials. Therefore, treatment decisions are usually made based on clinical experience, expert's opinions and clinical cases. The major drugs, including glucocorticosteroids, cyclosporine A (CsA) and etoposide (VP16), are usually used to initially treat and rapidly control hyperinflammation and hipercytokine response according to the protocols of HLH-2004. However, as for patients with central nervous system (CNS) involvement, dexamethasone (Dex) is the preferred corticosteroid because it can cross the blood-brain barrier better than prednisone or prednisolone. Although cyclosporine is the most frequently used immunosuppressive drug in HLH patients associated with autoimmune diseases [9], it remains controversial whether it is suitable for M-HLH patients.

Malignancy-directed and HLH-directed therapies are frequently overlapped. In LAHS patients, an etoposide-based chemotherapy regimen should be strongly considered [61]. Etoposide can inhibit topoisomerase II, thus leading to the breakage of double-stranded DNA. Meanwhile, etoposide can selectively deplete activated T cells to suppress the production of inflammatory cytokines and improve the survival in a murine model of HLH [62]. A study has reported that the mortality rate is increased by 14 times in children with EBV-associated HLH if etoposide is not provided within the first 4 weeks [63]. Other reports in adult cases have also indicated that regimens containing etoposide are associated with better survival [9]. In circumstances with EBV infection or unknown genetic predisposition, an etoposide-based regimen should be initiated without delay, which targets to immune dysregulation, EBV infection and malignancies.

There are very limited data on the treatment of C-HLH. Whether HLH-directed regimens are optimal for these patients depends on the severity of HLH and the possible triggers. Infections resulted from chemotherapy-induced immunosuppression are major triggers in C-HLH patients. The major triggers include viruses, invasive fungi and bacteria. Therefore, if infections occur in C-HLH patients, postponing chemotherapy or interruption of maintenance therapy should be considered, except for neoplasm relapse. Glucocorticosteroids and immunoglobulins can be administered to HLH patients [64, 65]. If possible, anti-infectious prophylaxis and powerful anti-infective treatment should be strongly considered in active HLH patients. Whether more aggressive treatment, such as etoposide, is beneficial for these patients still remains largely unexplored [65].

Anti-viral treatment should be performed if an obvious viral trigger is found, such as EBV, CMV or adenovirus. It has been reported that rituximab-containing immunochemotherapy benefits EBV-related HLH or malignancy patients with EBV infection [64]. Usually, rituximab can eliminate EBV-infected B cells, thus effectively playing an anti-virus role. However, EBV ectopically infects T or NK cells in majority of HLH patients in Asian countries, therefore, a combination of Dex/etoposide (VP16) or CsA controls EBV-infected T cells or NK cells. Alemtuzumab, an anti-CD52 monoclonal antibody, can also be used in the treatment of EBV-infected T cells or to destroy mature lymphocytes in T-cell lymphoma.

Stem cell transplantation (SCT), including allogeneic SCT, matched unrelated donor SCT and reduced-intensity conditioning (RIC) SCT, has revolutionized the treatment and resulted in long-term survival even cure in part of LAHS patients. However, bone marrow transplantation should be avoided in individuals with active disease because of cytokine storm and increased risk of graft-versus-host disease.

Standard therapy shows an obviously improved survival rate of HLH patients, while approximately 30% patients unresponsive to standard therapy are called as refractory HLH patients. So far, only few studies have focused on refractory HLH. Patients who do not achieve at least partial response 2 or 3 weeks after initial standard HLH therapy should be classified as refractory HLH [65, 66]. Effective salvage therapy can control refractory HLH to acquire time and chance for further therapy, thus prolonging the survival of the patients. A multi-center prospective study has analyzed the data from 63 adult HLH patients after DEP regimen (liposomal doxorubicin, etoposide and methylprednisolone) as a salvage treatment, a total of 29 patients are diagnosed as LAHS, and the overall response rate is 75.7% [66]. In addition, alemtuzumab as salvage therapy has been reported to improve the survival and achieve more time for allogeneic SCT in some refractory HLH patients. In EBV-related HLH patients with or without lymphoma, rituximab can be an option of salvage treatment [67]. Other novel drugs, such as infliximab, daclizumab, anakinra, vincristine and tocilizumab, have been also reported to apply in the refractory HLH patients [68]. Recently, another interesting development has been seen that thalidomide enhances the release of IL-2 and IFN from activated T cells, inhibits the immunosuppressive activity of regulatory T cells and increases NK-cell mediated cytotoxicity, thus inhibiting the release of TNF and other cytokines, including IL-6. However, it is still difficult to acquire reliable data from M-HLH because these reports are mainly based on case reports or other types of HLH [69].

### Prognosis

HLH is one of the most critical clinical disorders with a mortality rate of approximately 41% [9]. The survival data from several studies have shown that approximately 56–70% patients have a median overall survival of 36–230 days, and the 3-year survival of M-HLH patients is 18–55% [9]. Moreover, T-cell lymphoma-triggered HLH has a worse prognosis than B-cell lymphoma-triggered HLH. In a Japanese study, the 5-year overall survival rate in HLH patients with T/NK-cell lymphoma is only 12%, while it is 48% in HLH patients with B-cell lymphoma [20, 22, 23, 70]. Prognostic factors associated with mortality include thrombocytopenia, high onset age, possible lymphoma, increased ferritin levels, low Fbg level and EBV-DNA > 1,000 copies [17, 71]. Our previous retrospective analysis has indicated that active EBV infection, malignancy, low Fbg, low PLT and high LDH levels can predict high death risk and very poor prognosis [3, 24]. There are different prognostic factors for M-HLH and C-HLH. Underlying neoplasia or/and EBV infection are major prognostic factors in M-HLH patients, while infections, especially EBV infection and invasive fungal infection, may be the major factors in C-HLH patients. Other predictive factors, such as decreased number of CD3+ cells, have been reported to be adversely associated with survival of HLH patients [70]. Low glycosylated ferritin concentration is a predictive marker of severe HLH [71]. Moreover, bone marrow increased 18F-2-fluoro-2-deoxy-D-glucose (FDG) uptake on PET/CT may be a promising predictor for the extent of cytokine storm and the overall survival of LAHS patients [72]. The sensitivity, specificity and diagnostic accuracy of PET/CT for malignancy detection are 83, 62.5 and 71.4 %, respectively [30].

## CONCLUSIONS

Taken together, M-HLH is an increasingly recognized but life-threatening disease, which is associated with rapidly deteriorated clinical process and very high mortality. Currently, there is little evidence for M-HLH, especially for the diagnosis and therapeutic approach. With increased understanding of M-HLH, multidisciplinary collaboration is urgently required to promote international multicenter prospective study and develop clinical guidelines, thus finally improving the overall survival of M-HLH patients.
